# Comparison of intravitreal ranibizumab monotherapy vs. ranibizumab combined with dexamethasone implant for macular edema secondary to retinal vein occlusion

**DOI:** 10.3389/fmed.2022.930508

**Published:** 2022-09-12

**Authors:** Xuemei Liang, Baiyun Shen, Zuguo Ou, Hongmei An, Li Li

**Affiliations:** Nanning Aier Eye Hospital, Nanning, China

**Keywords:** retinal vein occlusion, macular edema, ranibizumab, dexamethasone implant, efficacy and safety

## Abstract

**Purpose:**

To compare the efficacy and the injection number of intravitreal ranibizumab (IVR) monotherapy vs. intravitreal ranibizumab plus dexamethasone (IVR + DEX) implants for macular edema (ME) secondary to retinal vein occlusion (RVO).

**Methods:**

This prospective, control trial comprised 96 eyes of 96 patients with ME due to non-ischemic RVO divided into two groups. The IVR monotherapy group consisted of 61 patients (29 with CRVO and 32 with BRVO) treated with ranibizumab with three consecutive loading doses at a monthly + pro re nata (three + PRN) regimen. The IVR + DEX implant group consisted of 35 patients (19 with CRVO and 16 with BRVO) treated with intravitreal ranibizumab plus DEX implant. All eyes underwent best-corrected visual acuity (BCVA, log MAR), central foveal thickness (CFT), and intraocular pressure (IOP). In case of recurrence, each group received initial medication.

**Results:**

At the 12-month visit, the mean log MAR BCVA that was improved from baseline was 0.23 with the IVR group and 0.30 with the IVR + DEX group. CFT decreased on average by 420 ± 292 μm with the IVR group and 393 ± 259 μm with the IVR + DEX implant group. No significant differences were detected in BCVA improvement and CFT reduction between the two groups (*p* > 0.05). The mean number of injections was 5.4 in the IVR group and 3.9 in the IVR + DEX implant group (*p* < 0.001). The mean reinjection interval for patients with the IVR + DEX implant was 131.2 ± 8.9 days (range: 98–150). The incidence of high IOP and cataract progression were significantly higher in the IVR + DEX implant group than in the IVR group (both *p* < 0.001).

**Conclusion:**

In RVO-ME, the IVR + DEX implant did not have synergistic efficacy, providing further improvement in BCVA and a reduction in CFT. However, the IVR + DEX implant still had an advantage in reducing the number of injections and prolonging the time between injections.

## Introduction

Macular edema (ME) secondary to retinal vein occlusion (RVO) is the leading cause of vision loss. In 2015, the global prevalence of RVO in people aged 30–89 years was 0.77%, equivalent to an overall 28.06 million worldwide (1). The pathogenesis of RVO involves an increase in inflammatory mediators and the upregulation of vascular endothelial growth factor (VEGF) that contributes to vascular leakage, the breakdown of the blood-retinal barrier, and ME (2, 3). Anti-inflammatory and anti-angiogenic pharmacotherapies have been developed for ME. Since 2010, intravitreal anti-VEGF agents, such as ranibizumab (Lucentis^®^; Genentech Inc., San Francisco, CA, United States), have been approved for the treatment of RVO-related ME and are widely applicable (4–6). Recently, dexamethasone (DEX implant; Ozurdex^®^; Allergan Inc., Irvine, CA, United States) intravitreal implant has become popular and has been increasingly used to treat RVO-related ME, which is the only indication for DEX in China. It has also been proven to be an effective therapy for RVO-related ME with a favorable long-term safety profile (7, 8). However, none of them are effective for all patients and have limitations. Ranibizumab is one of the fast-onset anti-VEGF agents with short-term effects, accompanied by frequent injections (9, 10). This casts a substantial burden on patients and poses a risk of complications (5, 11). As a sustained-release drug, the implant could secrete low doses of DEX into the vitreous cavity over a period of 6 months (12). Nonetheless, the DEX implant is not effective in all patients. Consequently, complementary and alternative treatments, such as combination therapy, are expected to address the multifactorial pathophysiological aspects of this disease and provide fast-onset and favorable long-term functional and anatomical improvements.

Herein, we introduced a clinical treatment pathway to compare the efficacy and the injection number of anti-VEGF using ranibizumab monotherapy vs. ranibizumab combined with DEX implant for RVO-related ME and to determine if the IVR + DEX implant can be synergistic, providing further improvements in best-corrected visual acuity (BCVA) and central foveal thickness (CFT) reduction when compared with IVR alone. To standardize and optimize our local RVO treatment strategy, we described a 12-month follow-up experience on the real-world efficacy and safety data.

## Subjects and methods

### Subjects

In this prospective, consecutive, control trial, 96 eyes of 96 patients with ME secondary to non-ischemic RVO were recruited between January 2019 and December 2020 at the Nanning Aier Eye Hospital (GuangXi, China) and studied over a 12-month follow-up period. The inclusion criteria were as follows: patients at least 18 years old and eyes that had not received any intravitreal injection for ≥ 6 months. In addition, the baseline BCVA (logMAR) was between 0.3 and 1.3. The exclusion criteria were as follows: ischemic RVO, diabetes mellitus, glaucoma, patients treated with macular laser previously, and those associated with other fundus diseases that affect vision. This study was approved by the Ethics Committee of Aier Eye Hospital, and all patients signed a written informed consent.

### Methods

**FIGURE 1:**
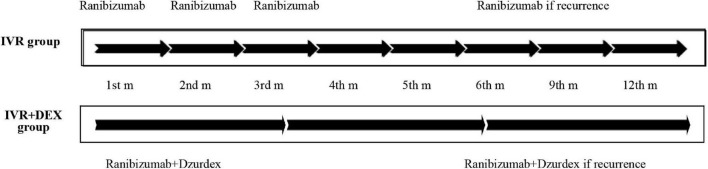
Treatment strategy (timeline): ranibizumab (IVR) monotherapy vs. ranibizumab plus dexamethasone (IVR + DEX) implant. During the study period, recurrence was treated in both groups at CFT > 300 μm or presenting subretinal or intraretinal fluid.

The cohort of 96 patients was divided into two groups. The IVR group, including 61 eyes (29 with CRVO and 32 with BRVO), was treated with three consecutive loading monthly doses of ranibizumab + pro re nata (three + PRN) regimen. Ranibizumab was continued in the event of ME recurrence. The IVR + DEX implant group, including 35 eyes (19 with CRVO and 16 with BRVO), was treated with intravitreal ranibizumab plus DEX implant from the beginning. In the case of recurrence, combination therapy was continued (Figure 1). Patients were eligible for retreatment if CFT was > 300 μm or if they presented with subretinal or intraretinal fluid.

BCVA was measured using the logarithm of the minimum angle chart visual acuity, and CFT served as the quantitative measurement of ME using optical coherence tomography (OCT) (Spectralis-OCT, Heidelberg Engineering, Germany). IOP was measured using the CT80A non-contact pneumatic tonometer (Topcon, Japan) and wide-angle scanning laser ophthalmoscopy (SLO) (panoramic 200Tx, OPTOS, United Kingdom). Fluorescein angiography was performed initially and after 6 months. Clinical evaluation was performed at baseline and at 1, 2, 3, 4 5, 6, 9, and 12 months after initiating therapy.

The primary clinical outcome measures were BCVA, CFT, and the number of injections in 12 months. The secondary outcome measures included the peak changes in BCVA, CMT from baseline, the reinjection interval, the percentage of patients with IOP > 21 mmHg, the percentage of patients who underwent cataract progression, and the percentage of patients who changed to ischemic at 6 months.

### Data collection

The clinical data of the eligible patients were recorded. Baseline demographics, including age, gender, and duration of symptoms, were collected. Ophthalmic data at baseline were the type of RVO, lens state (phakic and pseudophakia), BCVA, and CFT. Ophthalmic data at each visit, including BCVA, CFT, IOP, number of injections, the reinjection interval (days), degree of lens opacity, and postoperative complications, were recorded. The reasons for loss to follow-up were also noted.

### Data analysis

All statistical analyses were performed using IBM SPSS Statistics version 22 (IBM Corp., Armonk, NY, United States). Graphs were plotted using GraphPad Prism (version 8.0c, GraphPad Software, Inc.) and Adobe Illustrator CS 6. The mean ± SD is presented for the data that meet the normal distribution. The median ± interquartile range (IQR) was presented for the non-normal distribution data. Categorical data were analyzed using the Pearson χ^2^ test. Continuous variables and normal distribution data were analyzed using Student’s *t*-test and the Wilcoxon rank test, respectively. Changes in BCVA and CFT at various time points from baseline were tested using the Pair Wilcoxon signed-rank test. Statistical significance was set at a *p*-value < 0.05. All the tests were two-sided.

## Results

### Demographics and baseline characteristics

Table 1 describes the baseline demographics and characteristics of the 96 patients with unilateral eye involvement. The demographics and baseline characteristics did not vary significantly between the two groups (*p* > 0.05).

**TABLE 1:** Demographics and baseline characteristics.

	Total (*n* = 96)	IVR group (*n* = 61)	IVR + DEX Implant group (*n* = 35)	*p-value*
Age, years				
Mean (SD)	58.3 (12.7)	60.9 (11.1)	55.7 (14.1)	0.17
Range	19–83	39–83	19–76	
Sex, n (%)				0.14
Female	40	29	11	
Male	56	32	24	
Duration, days				0.09
Mean (SD)	61.4 (69.4)	68.1 (75.2)	49.7 (57.1)	
Range	1–365	3–365	1–180	
RVO, n (%)				0.53
CRVO	48	29	19	
BRVO	48	32	16	
Mean BCVA (SD)	0.79 (0.34)	0.79 (0.33)	0.77 (0.37)	0.66
Mean CFT, mm (SD)	658.9 (275.8)	683.1 (290.9)	616.8 (245.8)	0.23
Phakic	55	36	19	0.73

SD, standard deviation; CRVO, central retinal vein occlusion; BRVO, branch retinal vein occlusion; BCVA, best-corrected visual acuity; CFT, central foveal thickness.

### Visual outcomes and subgroup analyses

**FIGURE 2:**
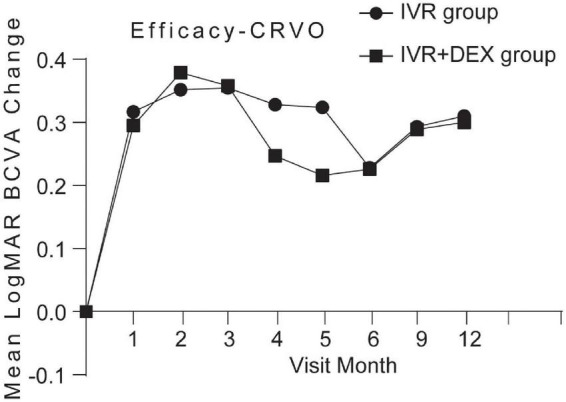
Mean log MAR BCVA improvement over time compared to baseline: 12-month follow-up in both treatment groups after initial therapy in patients with CRVO.

**FIGURE 3:**
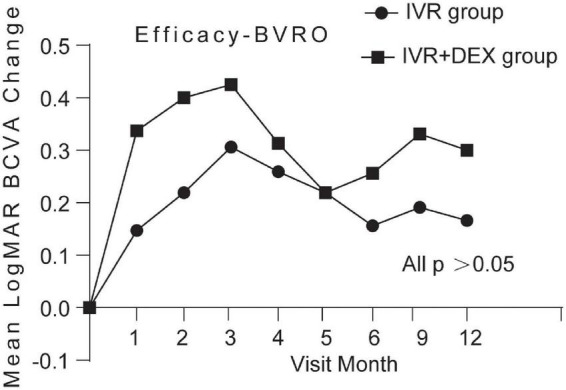
Mean log MAR BCVA improvement over time compared to baseline: 12-month follow-up in both treatment groups after initial therapy in patients with BRVO.

Mean log MAR visual acuity at the 12-month visit improved from baseline, by 0.23 with the IVR group and 0.30 with the IVR + DEX group. The percent of patients who gained 3 lines was 77.1% (47/61) and 82.8% (29/35), respectively. However, no significant differences were detected in BCVA improvement between the two groups (*p* > 0.05). When each group was further subdivided according to the types of RVO. Table 2 and Figures 2, 3 show the mean change of log MAR visual acuity from baseline for patients with CRVO and BRVO. Similarly, subgroup analysis showed no significant improvement of BCVA in the IVR + DEX implant group compared to the IVR group at each visit time (all *p* > 0.05). For patients with CRVO, the mean peak BCVA improvement at 1 month was 0.5 and 0.3 at 2 months in the IVR group vs. the IVR + DEX implant group, respectively. For patients with BRVO, the mean peak improvement in BCVA was seen at 3 months: 0.3 in the IVR group vs. 0.25 in the IVR + DEX implant group.

**TABLE 2:** Log MAR BCVA improved and CFT decreased between the two groups.

Time/Parameter (Mean ± SD)	IVR group *N* = 61		IVR + DEX implant group *N* = 35	
Mean log MAR BCVA improved	CRVO patients *n* = 29	BRVO patients *n* = 32	CRVO patients *n* = 19	BRVO patients *n* = 16
1 month	0.32 ± 0.18	0.15 ± 0.27	0.29 ± 0.21	0.34 ± 0.32
2 months	0.35 ± 0.31	0.22 ± 0.18	0.38 ± 0.25	0.40 ± 0.26
3 months	0.36 ± 0.31	0.31 ± 0.24	0.36 ± 0.27	0.43 ± 0.30
4 months	0.33 ± 0.33	0.26 ± 0.32	0.25 ± 0.31	0.31 ± 0.32
5 months	0.32 ± 0.29	0.22 ± 0.29	0.22 ± 0.29	0.22 ± 0.29
6 months	0.23 ± 0.27	0.17 ± 0.29	0.23 ± 0.37	0.26 ± 0.34
9 months	0.29 ± 0.33	0.19 ± 0.28	0.29 ± 0.29	0.33 ± 0.29
12 months	0.31 ± 0.27	0.17 ± 0.31	0.30 ± 0.25	0.30 ± 0.33
Mean CFT decreased (μm)				
1 month	308 ± 337	355 ± 309	324 ± 211	281 ± 179
2 months	401 ± 348	328 ± 334	403 ± 245	267 ± 113
3 months	378 ± 386	317 ± 332	414 ± 261	321 ± 136
4 months	323 ± 331	346 ± 295	243 ± 231	357 ± 251
5 months	375 ± 319	343 ± 293	323 ± 279	248 ± 216
6 months	314 ± 338	291 ± 344	276 ± 398	269 ± 280
9 months	348 ± 341	373 ± 323	346 ± 310	343 ± 279
12 months	452 ± 295	391 ± 352	435 ± 264	344 ± 281

SD, standard deviation; CRVO, central retinal vein occlusion; BRVO, branch retinal vein occlusion; BCVA, best-corrected visual acuity; CFT, central foveal thickness.

### Central foveal thickness outcomes and subgroup analyses

**FIGURE 4:**
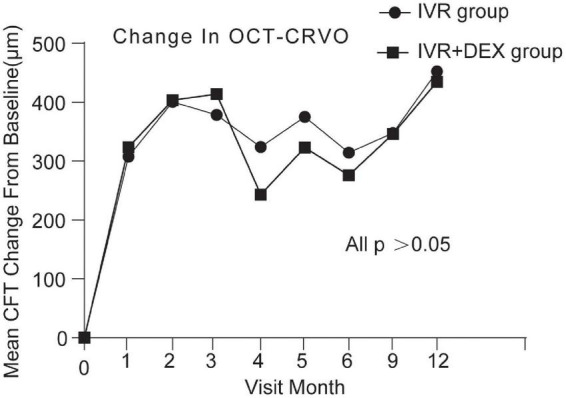
CFT decreased over time compared to baseline: 12-month follow-up in both treatment groups after initial therapy in patients with CRVO.

**FIGURE 5:**
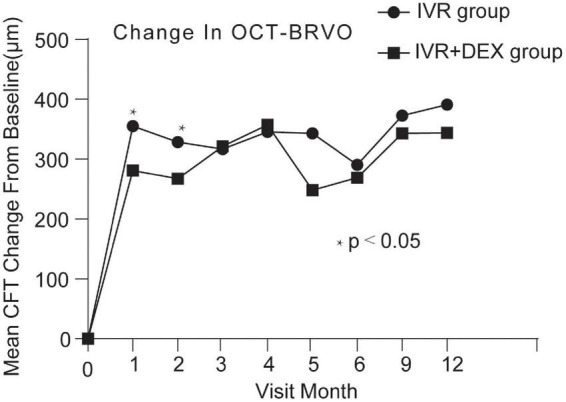
CFT decreased over time compared to baseline: 12-month follow-up in both treatment groups after initial therapy in patients with BRVO.

At the 12-month visit, CFT decreased on average by 420 ± 292 μm with the IVR group, and 393 ± 259 μm with the IVR + DEX implant group. The number of eyes achieving CFT < 250 μm was 43 (70.5%) and 26 (74.3%), respectively. When each group was further subdivided according to the types of RVO. Mean CFT decreases are shown in Table 2 and Figures 4, 5. For patients with CRVO, the IVR + DEX implant group showed no significant reduction in CRT compared to the IVR group (all *p* > 0.05). For patients with BRVO, the IVR group only showed a significant decrease in the first 2 months when compared to the IVR group (*p* = 0.07 and 0.09). A mean peak decrease was noted in CFT at 1 month, and a mean decrease of 364 μm in the IVR group vs. 307 μm in the IVR + DEX implant group was observed.

### Number of injections and reinjection interval

**FIGURE 6:**
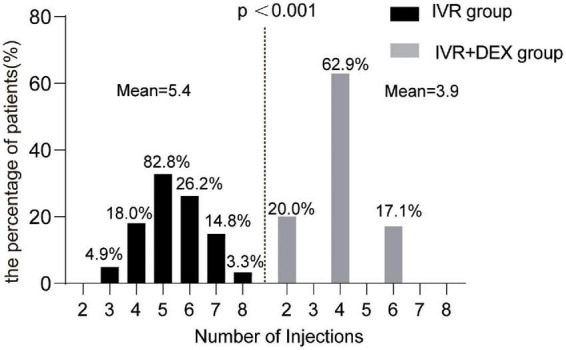
Number of injections received by RVO subgroups in 12 months.

Figure 6 shows the number of total injections received in the RVO cohort in 12 months. The mean number of total injections was 4.9 (range: 2–8). The mean number of injections of the IVR and the IVR + DEX implants group was 5.4 ± 1.2 (range: 3–8) and 3.9 ± 1.2 (range: 2–6), respectively. The mean number of injections was significantly fewer in the IVR + DEX implant group than in the IVR group (*p* < 0.001).

The mean total reinjection interval for all patients was 131.2 ± 8.9 days. The mean reinjection interval of CRVO and BRVO was 130.4 ± 10.2 (range: 98–145 days) and 134.6 ± 9.5 (range: 119–150 days), respectively. No statistically significant difference was detected in the mean intertreatment interval between the eyes with CRVO and BRVO (*p* = 0.15).

### Adverse events and associated procedures

An increase in IOP was defined as an IOP > 21 mmHg that lasted for > 3 days. The incidence of cataract progression and high IOP was significantly higher in the IVR + DEX implant group than that in the IVR group (*p* < 0.001). A high IOP in the eyes was controlled by topical drugs. All patients were non-ischemic at the baseline, and 6.2% of eyes (6/96) changed to ischemia at 6 months. There were 4 eyes in the IVR group and 2 eyes in the IVR + DEX group. However, no significant difference was detected between the two groups. For patients with ischemic RVO, laser photocoagulation and even grid photocoagulation were performed. Also, no neovascularization was noted at the posterior or anterior segment of the eye, and no other adverse events, such as intravitreal hemorrhage or endophthalmitis, were noted (Table 3).

**TABLE 3:** Adverse effects and other ocular procedures during the study period.

	Total *n* = 96	IVR group *n* = 61	IVR + DEX implant group *n* = 35	*p-value*
IOP > 21 (mmHg), n (%)	14 (14.6%)	1 (1.6%)	13 (37.1%)	< 0.001
Cataract progression	8 (14.5%)	0	8 (42.1%)	< 0.001
Non-ischemic changed into ischemic, n (%)	6 (6.2%)	14 (6.5%)	12 (5.7%)	0.87

## Discussion

Recently, a large number of studies have compared the efficacy and safety between anti-VEGF agents and DEX implants in patients suffering from RVO-related ME (13). Most studies demonstrated a relative superiority of anti-VEGF drugs in terms of visual acuity for patients with CRVO and BRVO, albeit not significantly (14–16). Currently, the idea of combination therapy for RVO-related ME is being promoted by ophthalmologists. However, studies based on the combination of anti-VEGF agents and DEX implants are scarce, and most lack a control group (17–20). In the present study, we designed a new treatment paradigm and control group, which has not yet been investigated.

In this study, we compared 1-year data on the functional and anatomical outcomes and the required number of injections of the IVR monotherapy and the IVR + DEX combination therapies initially for ME associated with RVO. Both treatments significantly contributed to BCVA improvement and CFT reduction in ME due to patients with RVO throughout the observation period. Nonetheless, no differences were detected in BCVA improvement between IVR monotherapy and IVR + DEX implant combination therapy. When each group was further subdivided according to the types of RVO, we found that IVR monotherapy can significantly decrease the CFT in BRVO patients in the early stage. However, in the late stage of patients with BRVO and patients with CRVO, no difference was observed between the two treatment regimens. It might mean that the DEX implant did not seem to have a combined effect with ranibizumab for eyes with RVO-ME. The lack of the same combination of paradigm and control group used in the earlier study made it difficult to compare these results to the findings of the previous studies.

In a prospective non-randomized case series, Harb et al. (20) evaluated the efficacy of the combination therapy of 2 mg of intravitreal aflibercept (Eylea^®^) and DEX implant, followed 2 weeks later, vs. DEX alone, and their study found that aflibercept with DEX implants achieved better visual outcomes. However, the study’s limitation was the lack of comparison based on the RVO subtype. Further, CRVO and BRVO are different disease entities. CRVO is prone to extensive ischemia, neovascularization, and blindness, and its natural history is poor compared to patients with BRVO (21). The current results may indirectly suggest that VEGF is the primary mechanism of RVO, ever combination with anti-inflammation therapy, patients with RVO did not have much better functional and anatomical outcomes. Hee et al. reported that BRVO is associated with elevated levels of interleukin (IL)-6, IL-8, and IL-17, especially VEGF (22). Testing the inflammatory cytokine and VEGF levels in the aqueous humor may provide an answer. However, our patients were not subjected to routine aqueous humor testing for cytokines.

For patients with CRVO, the mean peak BCVA improvement post-IVR monotherapy and IVR + DEX implant occurred at 1 and 2 months, respectively. Consequently, anti-VEGF agents showed a fast-onset, such that IVR could achieve a rapid BCVA improvement. These findings were similar to those observed in the CRYSTAL study group (9), a 24-month, prospective, open-label, single-arm, multicenter study to assess the 12-month efficacy and safety of 0.5 mg ranibizumab in patients with ME secondary to CRVO. The GENEVA study, a 6-month randomized, controlled clinical trial, followed by an additional open-phase 6-month trial, demonstrated a peak effect of DEX after 2 months and a progressive decline to baseline values at 6 months (7, 23).

In this study, we expected that the IVR + DEX implant would reduce the number of injections. The clinical setting results were as expected, and the number of injections was fewer in the IVR + DEX implant therapy than in IVR monotherapy. This was a substantial advantage of the IVR + DEX implant over the IVR. Treatment with an IVR + DEX implant could result in only two or three injections a year, which was lesser than the IVR injections required (24). Moreover, combination therapy reduced the burden on patients due to an intensive injection regimen and the requirement for multiple hospital visits (25).

The injection frequency influenced the treatment efficacy in both CRVO and BRVO. The mean intertreatment interval was 134 days in the present study. The mean reinjection interval was similar in eyes with CRVO and BRVO. Singer et al. (18, 26) conducted two interventional case series studies in which patients receiving DEX implants 2 weeks after anti-VEGF injections had a mean reinjection interval of 135 days and 126 days, respectively. Under the approved dose regimen, protocols administering DEX according to an as-needed retreatment protocol at 6-month intervals showed significantly lower efficacy (16). The recurrence of ME was observed by 4.5 months in the IRV + DEX group. The European Society of Retina Specialists recommended that the DEX implant should be readministered at 4–5 month intervals to maintain efficacy (27). Hence, emphasis and attention should be directed to real-world trials since the treatment intervals approach 4 months.

Concerning the safety profile, raised IOP and increased risk of cataract in phakic eyes by DEX were unavoidable (28). The GENEVA trial (23) through two identical, multicenter, and prospective studies suggested that, over 12 months, cataract progression occurred in 29.8% of patients. Singer et al. (18) conducted an open-label, interventional, case series but lacked a control group, showing that 26/44 (59%) phakic eyes that were given anti-VEGF therapy plus DEX therapy underwent cataract surgery. In the current study, 42.1% of phakic eyes in the IVR + DEX implant group experienced cataract progression after the second injection. The incidence varied greatly, which could be attributed to the duration of follow-up and the number of injections. The study of Lamprakis et al. showed that multiple (≥ 10) intravitreal anti-VEGF injections in one eye were not associated with an increased risk of sustained IOP-elevation (29). Previous studies on IOP varied greatly because of the definition of high IOP and the number of injections. The rates of IOP in the IVR + DEX implant were significantly higher than those in the IVR monotherapy (1.6% *vs*. 37.1%), with a peak at 2 months and similar to those in the anti-VEGF + DEX therapy (Harb *et al. 20.05%* (20), *Singer et al. 30.6%* (18), *and the GENEVA trial* (23), *12.6 and 15.4% after the first and second treatments, respectively).* IOP exceeding the normal range was controllable by topical IOP-lowering medication alone. The IVR treatment proved to be safer than the IVR + DEX treatment for adverse ocular reactions.

An evidence-based systematic review aiming to analyze the natural history of CRVO found that 15% and 34% of eyes with non-ischemic CRVO converted to ischemic CRVO over 4 months and 3 years, respectively (22). LEAVO study, a prospective, three-arm, double-masked, randomized non-inferiority trial, demonstrated a clinical diagnosis of conversion to an ischemic CRVO in 5.4% of patients who received anti-VEGF therapy for 100 weeks (30). In the present study, only 6.2% of eyes converted to ischemic RVO at 6 months. We speculate that the prevalence of neovascularization may be significantly masked during aggressive intravitreal therapy. However, the incidental effect of this treatment on the natural history of retinal non-perfusion is unclear (31).

There are three main limitations to the present study. First, it was affected by the novel coronavirus (2019-nCoV) outbreak in late 2019. It was difficult to maintain regular follow-up, and undertreatment is a frequent occurrence. Second, unlike the RCTs, the choice of therapy depends mainly on the patient’s economic condition and the convenience of follow-up. Third, as we are a specialized ophthalmic hospital and have limited physical examination equipment, we failed to investigate systemic abnormalities that might affect the therapeutic effect.

In conclusion, the current study showed that the combination of IVR and DEX implant had no superiority in BCVA improvement and CFT decreased when compared with IVR monotherapy. However, an IVR + DEX implant is still an option because it has fewer number of injections and could be maintained with retreatment every 4–5 months. The adverse effects, such as raised IOP and increased risk of cataracts in phakic eyes, were more pronounced with the IVR + DEX implant than with IVR monotherapy. Timely and aggressive treatment may significantly reduce the percent conversion of non-ischemic to ischemic.

## Data availability statement

The original contributions presented in this study are included in the article/supplementary material, further inquiries can be directed to the corresponding author/s.

## Ethics statement

The studies involving human participants were reviewed and approved by Ethics Committee of Aier Eye Hospital. The patients/participants provided their written informed consent to participate in this study.

## Author contributions

XL participated in study conception, writing the manuscript, dataset interpretation, and final revision. BS and ZO worked on figure artwork, tables, and statistics. HA helped with photographic material compilation. LL carried out final revision and writing of the conclusion. All authors contributed to manuscript revision, read, and approved the submitted version.
